# Impaired circadian heart rate variability in Parkinson’s disease: a time-domain analysis in ambulatory setting

**DOI:** 10.1186/s12883-020-01722-3

**Published:** 2020-04-23

**Authors:** V. Arnao, A. Cinturino, S. Mastrilli, C. Buttà, C. Maida, A. Tuttolomondo, P. Aridon, M. D’Amelio

**Affiliations:** 1 Department of Biomedicine, Neuroscience and Advanced Diagnostics (BiND), University of Palermo, Via Gaetano la Loggia n.1, 90129 Palermo, Italy; 2 Dipartimento Biomedico di Medicina Interna e Specialistica (Di.Bi.M.I.S), Università degli Studi di Palermo, Palermo, Italy

**Keywords:** Non-motor symptoms, Autonomic disorders, Heart rate variability, SCOPA-AUT

## Abstract

**Background:**

Heart rate variability (HRV) decreases in Parkinson’s disease (PD) and it can be considered a marker for cardiovascular dysautonomia. The purpose of this pilot study is to evaluate long-term time-domain analysis of HRV of PD patients and compare the results with those of matched healthy individuals.

**Methods:**

Idiopathic PD patients without comorbidity impairing HRV, and age-matched healthy individuals were recruited in a pilot study. A long-term time domain analysis of HRV using 24-h ambulatory ECG was performed.

**Results:**

Overall, 18 PD patients fulfilling inclusion criteria completed the evaluation (mean age was 55.6 ± 8.8, disease duration: 5.0 ± 4.7). Mean SCOPA-AUT score was 10.1 ± 7.3. Patients were on Hoehn & Yahr stage 1–2 and mean Levodopa Equivalent Dose (LED) was 311 ± 239.9. Mean of the 5-min standard deviation (SD) of R-R intervals distribution (SDNN) for all 5 min segments of the entire recording (ISDNN) was significantly lower in patients compared to controls. ISDNN was significantly different between Parkinson’s disease patients and healthy controls.

**Conclusions:**

In our population characterized by mild to moderate disease severity, time-domain assessment of HRV seemed to be a potential tool to characterize cardiovascular dysautonomia. Decrease of ISDNN in PD may reflect an autonomic derangement extending all day and night long.

## Background

Heart rate variability (HRV) is decreased in Parkinson’s disease (PD) and it could occur early [1] in the course of the disease. HRV can be considered a marker for cardiovascular sympathovagal balance [2]. HRV is a non-invasive widespread tool for studying heart rate regulation in relation to autonomic system. HRV might be evaluated by a short term (usually 5 min) ECG recording obtained under controlled standardized conditions and long term analysis [3]. Typically, higher values of HRV reflect better health and studies using HRV evaluation have reported that it declines in patients with PD compared to healthy matched controls, in short and long recordings [4, 5]. Short-time spectral analysis is often used in PD patients because it is a fast, low-cost and patient-independent measure, with good intra-individual reproducibility for HRV [3–6]. However, it has been observed that patients with normal responses to short-time spectral analysis tests could suffer from impaired HRV evaluated by a long-term time analysis [3]. Time domain analysis by a 24-h ECG recording could be a valid tool for characterizing cardiac autonomic state in PD and 24-h HRV indices appear to be stable and free of placebo effect [7].

The main aim of our study was to evaluate whether a difference between PD patients without comorbidities impairing long term time-domain HRV and healthy controls exists. Whether a difference was observed, we determined the characteristics of the disease associated with HRV impairment. A time-domain assessment of HRV was performed using a long term recording.

## Methods

### Characteristics of the population

This was an observational, cross-sectional, comparative pilot study carried-out in an ambulatory setting. Non-demented PD patients were consecutively recruited from our department. Patients with suspected atypical or secondary parkinsonism were excluded, as well as those with comorbidities known to influence HRV (hypertension, diabetes, heart diseases, heart failure, myocardial infarction), or those patients taking drugs that are known to reduce HRV (antidepressants, selegeline, anticholinergic, mineral corticoids, beta blockers, calcium blockers, antiarrhythmics). Similar exclusion criteria were used for healthy controls (individuals non affected by Parkinson’s disease or other neurological disorders), who were matched 1:1 for sex and age to PD patients. All the participants gave their informed consent to participate in the study. The study was approved by the local ethics committee (Palermo1 v.n.5/13.05.2015) and conducted according to the principles of the Declaration of Helsinki.

During the study period, 127 patients (males, 55%) were evaluated. Overall, 104 patients were excluded because they did not meet the inclusion criteria (22 demented, 82 for other comorbidities or taking drugs affecting HRV). Five of the final 23 patients invited, refused to participate in the study.

### Clinical and ECG evaluation

Each patient underwent an extensive evaluation including Hoehn & Yahr stage (H&Y) [8], Unified Parkinson’s Disease Rating Scale (UPDRS) [9], and Scale for Outcomes in PD for autonomic symptoms (SCOPA-AUT) [10]. Medications and comorbidities were recorded, using the Cumulative Illness Rating Scale (CIRS) [11]. Levodopa Equivalent Daily Dose (LED) [12] was also calculated. A long term time domain analysis of heart rate variability (HRV) using 24-h 12-lead ECG recording (at least 18-h ECG), encompassing morning and night hours was performed and evaluated according to the NASPE/ESC Task Force [7] in PD patients and age-matched healthy individuals. Both groups were asked to perform normal daily activity excluding intense physical activity. All time domain parameters analysed have been summarized in Table 1 and calculated using “cardioscan II” software (version 11.4.0054a).

**Table 1:** HRV Time domain parameters evaluated in PD patients and controls

Variables	Description (*unit*)
SDNN 24	Standard deviation (SD) of all NN intervals (*ms*)
SDNN D	
SDNN N	
pnn50 24	Percentage of successive RR intervals that differ by more than 50 ms (%)
pnn50 D	
pnn50 N	
rMSS 24	Root mean square of successive RR interval differences (*ms*)
rMSS D	
rMSS N	
SDANN	Standard deviation of the average NN intervals for each 5 min segment of a 24 h HRV recording (*ms*)
ISDNN	Mean of the Standard deviation of all NN intervals for all 5 min segments of the entire recording (ms)

*PD* Parkinson’s disease, *D* day, N: night

### Statistics

Results are expressed as mean ± SD, with *p* ≤ 0.05 considered significant. Analysis of normality was performed with the Shapiro-Wilk W test (α: 0,05). Differences between cases and controls of HRV time domain parameters were calculated by t-test analysis. When a variable of the HRV time domain analysis was found significantly different between cases and controls, its association with explored PD variables was evaluated by Pearson correlation. Furthermore, linear regression analysis investigated for correlations between patient characteristics: age, LED, HY, LEVODOPA, SCOPA-AUT (independent variables) and HRV time domain parameters (dependent variable) in simple and multiple regression models. B coefficients (B) and their 95% confidence intervals (CIs) were also calculated. The post-hoc statistical power and the effect sizes on the basis of standardized mean differences (Cohen’s d) were performed too by Clin.Calc.com (https://ckinical.com/stat/Power-aspx accessed on 01/12/2019) and G*Power 3.1.9.3 software to compute statistical power in sample size of our pilot study.

## Results

**Fig. 1:**
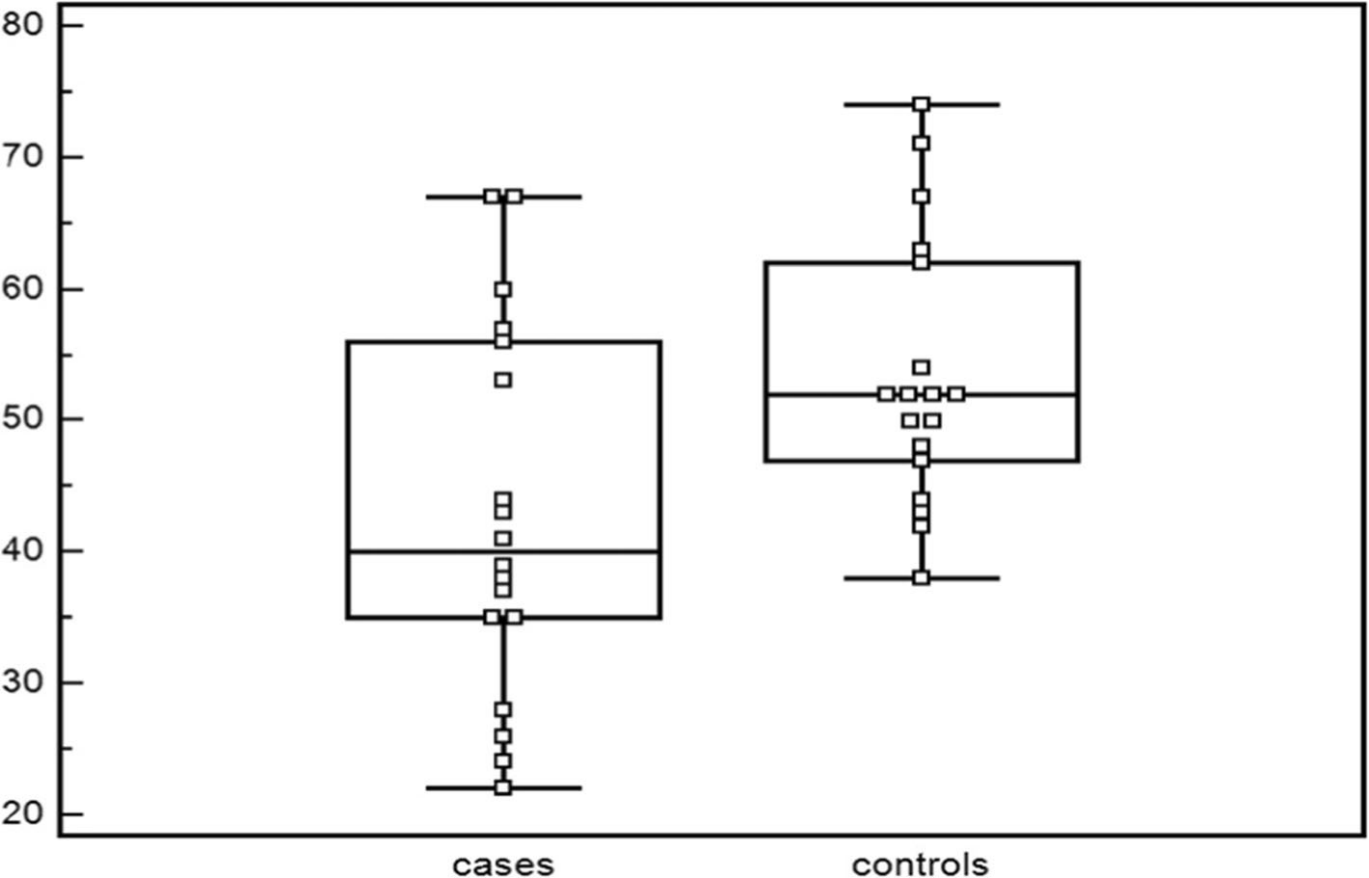
ISDNN mean and standard deviations in PD patients and controls

Overall, eighteen PD patients (9 males) with a mean disease duration of 50 ± 4.7 years were enrolled in this study. No significant difference of age at HRV evaluation was observed between PD patients (mean age 55.6 ± 8.8 years), and controls (mean age 56.0 ± 9.4 years) (*p* = 0.8). PD patients had a mean SCOPA-AUT scale score of 10.1 ± 7.3. Patients with PD were characterized by mild to moderate disease severity (HY stage 1–2) and mean LED was 311 ± 239.9. Only SDNN index (ISDNN), evaluating mean of the standard deviations of all NN intervals for all 5 min segments of the entire recording, was significantly different between PD patients (42.9 ± 14.2 ms) and healthy controls (53.4 ± 10.1 ms) (*p* = 0.01)(see Table 2). We plotted ISDNN values for PD patients and controls (see Fig. 1). Statistical power was calculated (power 72. 5%, α:0.05), whereas, observed Cohen’d was 0. 85.

**Table 2:** HRV parameters (means ± SD) in PD patients and controls

*Variables*	*PD patients (18)*	*Controls (18)*	*p*
*SEX(men%)*	*9(50)*	*9(50)*	
*AGE(years)*	*55.6 ± 8.8*	*55.6 ± 8.8*	*0.8*
*SDNN 24(ms)*	*122.6 ± 38.3*	*136.0 ± 21.4*	*0.2*
*SDNN D(ms)*	*84.3 ± 27.9*	*96.2 ± 36.5*	*0.3*
*SDNN N(ms)*	*112.6 ± 40.9*	*119 ± 31.3*	*0.6*
*pnn50 24(%)*	*4.5 ± 4.1*	*5.3 ± 5.3*	*0.6*
*pnn50 D(%)*	*2.5 ± 2.3*	*3.8 ± 4.8*	*0.3*
*pnn50 N(%)*	*9.3 ± 9.4*	*8.4 ± 7.6*	*0.8*
*rMSS 24(ms)*	*24.9 ± 7*	*25.9 ± 7.7*	*0.7*
*rMSS D(ms)*	*20.8 ± 5.5*	*23.4 ± 8.9*	*0.3*
*rMSS N (ms)*	*31.1 ± 11.9*	*29.6 ± 9.5*	*0.7*
*SDANN(ms)*	*118.1 ± 38.8*	*124.7 ± 21.2*	*0.5*
*ISDNN(ms)*	*42.9 ± 14.2*	*53.4 ± 10.1*	*0.01*

*PD* Parkinson’s disease; *D* day; *N* night

**Fig. 2:**
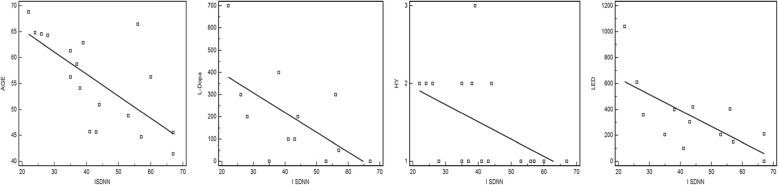
correlation by linear regression between ISDNN and clinical PD variables (AGE, Levodopa dosage, HY, LED)

ISDNN significantly correlated with age (*r* − 0.69, *p* = 0.02), LED (*r* − 0.60, *p* = 0.01), levodopa dosage (*r* − 0.57, *p* = 0.02) and SCOPA-AUT scale scores (*r* − 0.47, *p* = 0.05). At linear regression analysis in simple regression model, a statistically significant relationship between age, levodopa dosage, HY and LED and ISDNN (see Fig. 2) was observed, but not confirmed in multiple regression model.

## Discussion

In our study, recruiting only non-demented PD patients, with mild to moderate disease severity and no other conditions (comorbidities or treatments) known to affect HRV, ISDNN was significantly different between Parkinson’s disease patients and healthy controls. ISDNN seems to correlate with age, severity of disease stage, LED and levodopa dosages. Taking into account the task force on the HRV, SDNN, reflecting over heart variability, is not a well-defined statistical variable due to its dependence on ECG time recording, while ISDNN, evaluating too over heart variability, is a more standardized variable [7].

ISDNN most closely correlates with age, declining with aging; gender also influences HRV (women had lower values); however in our study we enrolled PD patients and healthy controls matched for age and sex [13].

In a recent study, including 1.741 PD patients (ECG recording has been available for 653 PD patients), no association was reported between HRV (SDNN and rMSS) and PD severity or its progression [14], although routine ECG data (standard 10-s 12-lead ECG) was used to perform HRV; standard ECG recording is considered inadequate for measuring HRV. According to the task force on the HRV, short-term 5-min recordings and nominal 24-h long-term recordings seem to be appropriate options. Moreover, patients with significant comorbidities or those taking drugs impairing HRV were not excluded. ISDNN, the mean of the 5-min standard deviation of the NN interval calculated over 24 h, which measures the variability due to cycles shorter than 5 min, requires a 24-h ECG recording. Both cardiac sympathetic and parasympathetic dysfunctions are commonly reported in PD [15] ISDNN primarily reflects autonomic influence on heart rate variability [16]. Decrease of ISDNN, reflecting over variability of HRV, may be demonstrated in PD by a sympathovagal balance impairment. A body of evidence has challenged the traditional view of PD as a motor disorder, in favour of the idea of PD being a more complex disorder, including non-motor symptoms [17]. Among non-motor symptoms, autonomic dysfunction is frequent in PD patients [18, 19] with cardiovascular autonomic dysfunction occurring early in the course of the disease [20]. ISDNN, evaluating HRV in 24 h, implies a more complex derangement extending all day and all night and it could reflect an impairment of diurnal-temporal component; suggesting an imbalance of circadian system as observed for other non-motor symptoms in PD. The balance between sympathetic and parasympathetic varies in synchrony with the circadian system [21]. Circadian dysfunction could be part of the new view of PD highlighting the role of non-motor symptoms and its temporal pattern. Our pilot study was characterized by strengths and limitations. The major strength of our study was the inclusion of individuals (patients and healthy controls) without comorbidities or taking drugs known to affect HRV. The observed difference in HRV between the two groups was therefore hypothetically almost exclusively due to the presence of PD. Other strengths of our study were the strict matching for age and sex between cases and controls, as well as the use of a validated scale, the SCOPA-AUT, in order to detect the presence of autonomic dysfunction. The main limitation of our pilot study was the small sample size, attributable to the decision to adopt rigid exclusion criteria including concomitant medical issues and the use of some drugs. This led to the elimination of nearly 65% of the patients. It is presumable that, in an ambulatory setting, autonomic evaluation would be in the near future considered together with motor and other non-motor examination. The evaluation should include a questionnaire such as the SCOPA-AUT scale, and tools such as the long-term time-domain assessment of HRV; as low-cost and fast evaluations have to be fostered, our findings suggest that, in an ambulatory setting, the use of HRV analyses could be considered as part of an instrumental evaluation for the detection of autonomic dysfunction. This is in fact a promising, simple, non-invasive and low-cost test for cardiovascular autonomic symptoms in PD patients.

## Conclusions

Autonomic symptoms are common but often unrecognized in patients with Parkinson’s disease. In our population characterized by mild to moderate disease severity, time-domain assessment of HRV seemed to be a potential tool for characterizing cardiovascular dysautonomia. In our pilot study, ISDNN was significantly different between PD patients and matched healthy controls. In an ambulatory setting, according to our findings, the use of HRV analysis could be considered as part of an instrumental evaluation for cardiovascular autonomic symptoms in PD patients.

## Data Availability

The datasets used and/or analysed during the current study are available from the corresponding author on reasonable request.

## Abbreviations

CIRS: Cumulative Illness Rating Scale
D: Day
HRV: Heart rate variability
H&Y: Hoehn & Yahr stage
ISDNN: Mean of the SD of all NN intervals for all 5 min segments of the entire recording
LED: Levodopa Equivalent Dose
N: Night
PD: Parkinson’s disease
pNN50: NN50 count divided by the total number of all NN intervals
r-MSSD: Square root of the mean of the sum of the squares of differences between adjacent NN Intervals
SCN: suprachiasmatic nuclei
SCOPA-AUT: Scale for Outcomes in PD for autonomic symptoms
SD: Standard deviation
SDNN: Standard deviation of all NN intervals
UPDRS: Unified Parkinson’s Disease Rating Scale
